# Resveratrol-like Compounds as SIRT1 Activators

**DOI:** 10.3390/ijms232315105

**Published:** 2022-12-01

**Authors:** Lidia Ciccone, Eugenia Piragine, Simone Brogi, Caterina Camodeca, Raffaele Fucci, Vincenzo Calderone, Susanna Nencetti, Alma Martelli, Elisabetta Orlandini

**Affiliations:** 1 Department of Pharmacy, University of Pisa, Via Bonanno 6, 56126 Pisa, Italy; 2 Center for Instrument Sharing, University of Pisa (CISUP), Lungarno Pacinotti 43, 56126 Pisa, Italy; 3 Department of Earth Science, University of Pisa, Via Santa Maria 53, 56126 Pisa, Italy; 4 Research Centre E. Piaggio, University of Pisa, 56126 Pisa, Italy

**Keywords:** resveratrol, sirtuin 1, SIRT1 activators, computer aided drug discovery, nature-inspired compounds, resveratrol-like compounds, structure-activity relationship (SAR), oxidative stress, vascular endothelium, endothelial dysfunction

## Abstract

The sirtuin 1 (SIRT1) activator resveratrol has emerged as a promising candidate for the prevention of vascular oxidative stress, which is a trigger for endothelial dysfunction. However, its clinical use is limited by low oral bioavailability. In this work, we have applied a previously developed computational protocol to identify the most promising derivatives from our in-house chemical library of resveratrol derivatives. The most promising compounds in terms of SIRT1 activation and oral bioavailability, predicted in silico, were evaluated for their ability to activate the isolated SIRT1 enzyme. Then, we assessed the antioxidant effects of the most effective derivative, compound **3d**, in human umbilical vein endothelial cells (HUVECs) injured with H_2_O_2_ 100 µM. The SIRT1 activator **3d** significantly preserved cell viability and prevented an intracellular reactive oxygen species increase in HUVECs exposed to the oxidative stimulus. Such effects were partially reduced in the presence of a sirtuin inhibitor, sirtinol, confirming the potential role of sirtuins in the activity of resveratrol and its derivatives. Although **3d** appeared less effective than resveratrol in activating the isolated enzyme, the effects exhibited by both compounds in HUVECs were almost superimposable, suggesting a higher ability of **3d** to cross cell membranes and activate the intracellular target SIRT1.

## 1. Introduction

Vascular oxidative stress is both a common feature and a major cause of age-related pathological disorders such as hypertension, type 2 diabetes, and atherosclerosis [1,2,3,4,5]. Over time, chronic oxidative stress can lead to the development of a sub-clinical and low-grade inflammatory state that further contributes to the onset of vascular dysfunction [6]. The primary target of vascular oxidative stress is the endothelium, as it is the first layer to be in contact with the blood flow. A wide range of circulating oxidative stimuli can directly damage the endothelium, thus leading to the loss of endothelial function, vascular hyperpermeability, and subsequent widespread oxidative and inflammatory markers in many organs and tissues [7]. Therefore, novel strategies aimed at protecting the endothelial layer from oxidative stimuli represent a current medical need.

In recent years, the potential use of many natural compounds in the prevention and treatment of vascular oxidative stress has been investigated [8,9]. Among them, polyphenols are the most promising candidates for phytotherapy and nutraceutical purposes. Many researchers have demonstrated the antioxidant and vasoactive properties of *Citrus* flavonoids, such as naringin and naringenin [10,11,12,13,14]. Furthermore, food supplements containing polyphenols (i.e., bergamot and orange juices) exhibited potential antioxidant effects in both pre-clinical and clinical studies [15,16,17]. Currently, the most characterized polyphenol is resveratrol, a stilbene mainly present in grapes and wine, which has been largely investigated in clinical and preclinical trials for its anticancer properties [18,19], its hypoglycemic activity [20], and also for its promising profile to improve the side effects of menstrual cyclicity [21].

Moreover, the antioxidant and vasorelaxant properties of resveratrol have been largely demonstrated [22,23,24,25,26,27]. Accordingly, nutraceutical products rich in resveratrol, such as grape pomace polyphenolic extracts, promote antioxidant and vasoactive effects in vascular cell cultures and hypertensive animals [28]. 

The mechanisms of action accounting for the antioxidant properties of resveratrol mainly involve the activation of sirtuin enzymes (SIRTs), a class of nicotinamide adenine dinucleotide (NAD^+^)-dependent histone deacetylases that play a pivotal role in the maintenance of cardiovascular homeostasis [29]. One of the most characterized in the cardiovascular system is isoform 1 (SIRT1), which is an epigenetic regulator of the endogenous antioxidant and anti-inflammatory defense systems. Indeed, SIRT1 promotes the expression of antioxidant genes (i.e., superoxide dismutase, SOD; catalase, CAT; glutathione peroxidase, GPx) via activation of the nuclear factor E2-related factor 2/antioxidant response element (Nrf2/ARE) pathway. Moreover, SIRT1 inhibits the transcription of pro-inflammatory genes via modulation of the nuclear factor kappa B (NF-κB) pathway [30,31]. As a central regulator of cell survival in response to detrimental stimuli, SIRT1 might play a relevant role in the development and progression of endothelial dysfunction, vascular ageing, and age-related cardiovascular diseases (CVDs). In fact, reduced expression of SIRT1 has been reported in patients with acute coronary syndrome, stable coronary artery disease, or a history of atrial fibrillation [32,33,34]. Therefore, SIRT1 activators have been proposed as potential candidates in the prevention and treatment of many CVDs associated with decreased SIRT1 activity, oxidative stress, and loss of endothelial function. 

In this regard, the activation of SIRT1 by resveratrol through direct binding to the enzyme [35] seems to be responsible for the protective effects promoted in various experimental models of oxidative stress [36,37]. Indeed, the antioxidant effects exhibited by this natural polyphenol in vivo were markedly reduced in SIRT1 knock-out animals [36] or in the presence of SIRT1 inhibitors [37,38]. However, clinical use of resveratrol is limited by both rapid metabolism and urinary excretion that lead to low oral bioavailability (less than 1% of the administered dose) [39,40]. To overcome such pharmacokinetic problems, new polyphenolic formulations have been proposed [41] and novel SIRT1 activators have been recently developed [42,43,44]. Among them, small molecules (i.e., SRT2104, SRT1460, SRT2183, and SRT3025) designed by Sirtris Pharmaceuticals (GlaxoSmithKline) have been involved in many clinical studies [45]. However, the most promising SIRT1 activator developed by Sirtris Pharmaceuticals, SRT2104, showed poor and highly variable oral bioavailability [46,47], thus stressing the need to develop synthetic derivatives exhibiting the same pharmacological properties of resveratrol but endowed with a more favorable pharmacokinetic profile to confer them a concrete translational potential in clinical practice [45]. 

**Figure 1:**
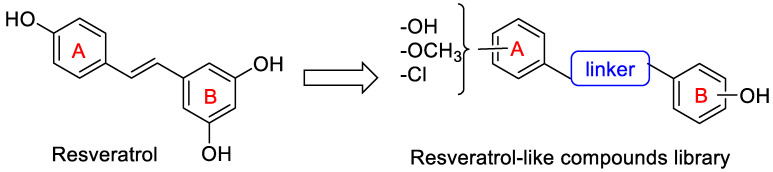
Chemical structure of resveratrol and general structure of the library of resveratrol-like compounds.

In this study, we have developed a computational protocol for identifying the most promising derivatives from our in-house chemical library in order to prioritize compounds to be tested in in vitro experiments able to act as SIRT1 activators, considering the affinity for SIRT1 enzymes, as well as the drug-like profile. The library is characterized by resveratrol-like compounds where the diaryl structure of resveratrol was maintained while the rigid alkene linker between the two aromatic portions was replaced by a more flexible methyleneoxyamine linker (Figure 1). Moreover, the compounds in the library present various types of substitutions on the two phenyl rings (A and B) to study the best combination for a suitable interaction with SIRT1.

**Figure 2:**
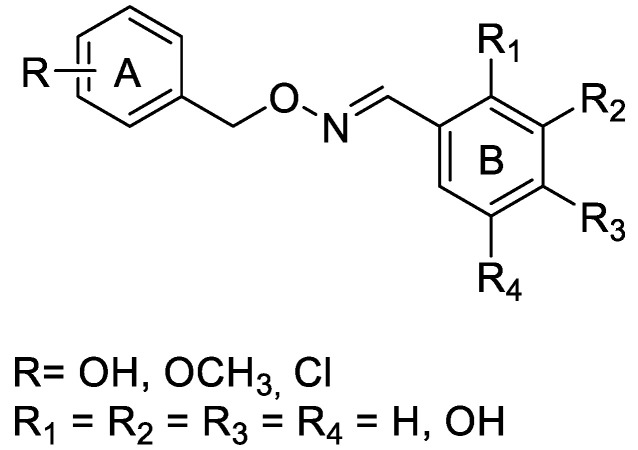
Chemical structure of the compounds in our in-house chemical library.

In addition to the obvious substitution of A and B rings with a hydroxyl group in the same position as those of resveratrol, the two aromatic rings were substituted with hydroxyl groups in various positions. In compounds **1–3**, the two phenyl rings were decorated with one hydroxyl group (ring A) or one/more hydroxyl groups in various positions (ring B), while in compounds **4–9**, the two phenyl rings were substituted with hydroxyl, methoxyl groups, or a chlorine atom in various positions (ring A) and one or more hydroxyl groups on ring B (Figure 2). The types of substituents on the A phenyl ring could indicate that when increasing the lipophilicity of the compounds, their penetration through membranes and tissues, including the blood–brain barrier, improves. 

Following our previously published computational strategy [43], we have selected six derivatives. Accordingly, to validate the in silico findings, we then assessed the ability of the most promising derivatives to activate the isolated SIRT1 enzyme using a cell-free approach. Among the screened compounds, the most effective SIRT1 activator was selected for further investigations in vitro. In particular, the potential preventive effects of the most promising compound against oxidative damage were assessed in human umbilical vein endothelial cells (HUVECs), both in the presence and absence of sirtinol, a well-known SIRT1 inhibitor.

## 2. Results

### 2.1. In-House Chemical Library of Resveratrol Derivatives

The compounds belonging to the chemical library are listed in Table 1. 

**Scheme 1:**
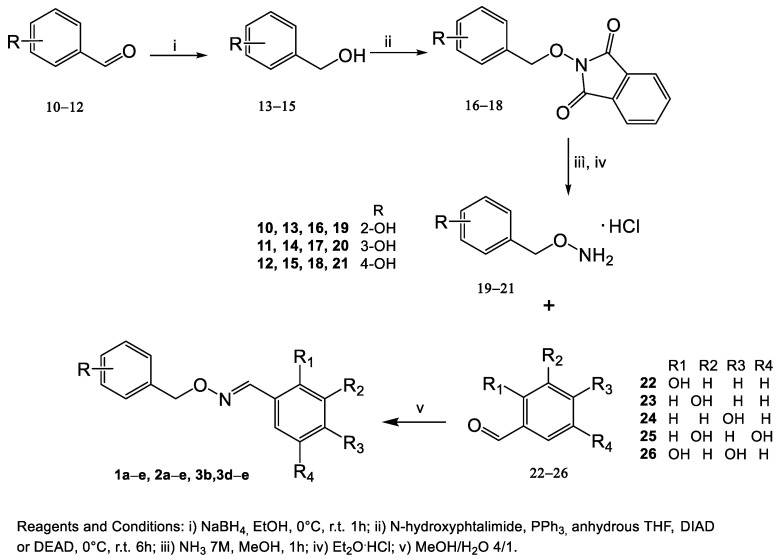
Synthesis of mono- or poly-hydroxylated (*E*)-benzaldehyde *O*-benzyl oxime derivatives **1a–e**, **2a–e, 3b**, **3d–e**.

The synthesis of (*E*)-mono- or poly-hydroxy substituted benzaldehyde *O*-(2- or 3- or 4-hydroxybenzyl)oxime derivatives (**1a–e**, **2a–e**, **3b**, and **3d–e**) started from commercially available hydroxybenzaldehydes as described in Scheme 1, while compounds **4a–e**, **5a–e**, **6a–e**, and **7–9** were resynthesized using the synthetic procedure previously described by us [48].

Upon reduction of the aldehydic group of **10–12** with sodium boron hydride in ethanol at room temperature for one hour, the alcohols **13–15** were obtained. The Mitsunobu reaction between the suitable intermediates **13–15** and *N*-hydroxyphthalimide in anhydrous tetrahydrofuran, triphenyl phosphine, and di-isopropyl-azo-dicarboxylate (DIAD) or di-ethyl-azo-dicarboxylate (DEAD) yielded the intermediates **16–18,** which were subjected to the removal of the protecting phthalimido group by treatment with ammonia solution 7 N in MeOH to give 2-, 3-, or 4-((aminooxy)methyl)phenols (**19–21**). Compounds **19–21** were transformed into the corresponding hydrochloride salts by adding ether hydrochloride.

Treatment of hydroxy-substituted *O*-benzylhydroxylamines **19–21** with the appropriate aldehydes **22–26** in a methanolic solution at room temperature afforded the desired mono- or poly-hydroxylated (*E*)-benzaldehyde *O*-benzyl oxime derivatives **1a–e**, **2a–e**, **3b**, and **3d–e** as the only geometric isomer. The correct configuration was attributed to their ^1^HNMR spectra in which the chemical shift value of the diagnostic iminic proton ranged from 8.41 to 8.00 ppm in accordance with the chemical shift values for the same proton reported by the literature [48,49].

### 2.2. In Silico Chemical Library Screening

**Figure 3:**
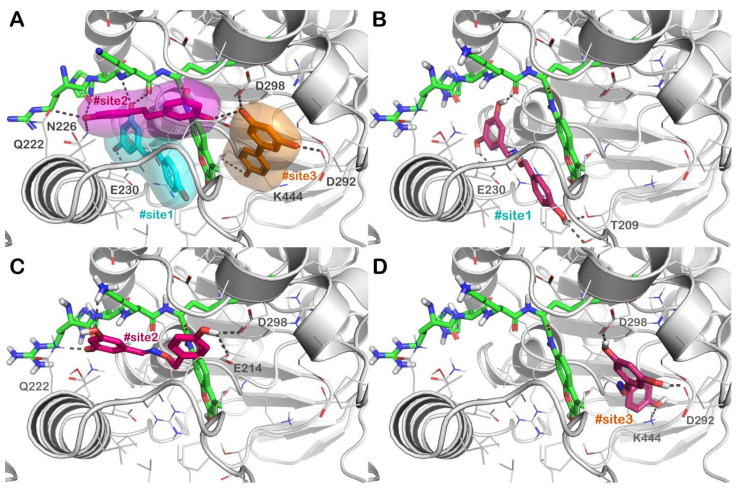
(**A**) Crystal structure of SIRT1 (grey cartoon representation) in complex with resveratrol (sticks representation) (PDB ID 5BTR); the different binding sites of resveratrol, namely, #site1, #site2, and #site3 are represented in cyan, magenta, and orange, respectively; (**B**–**D**) binding mode of compound **3d** (dark purple sticks) into SIRT1 with particular focus on #site1 (panel **B**), #site2 (panel **C**) and #site3 (panel **D**). The p53-AMC-peptide is reported as green sticks. Residues in the binding sites are represented by lines while hydrogen bonds are shown as grey dotted lines. Pictures were generated by PyMOL software (The PyMOL Molecular Graphics System, v1.8; Schrödinger, LLC, New York, NY, USA, 2015).

In order to identify the most promising derivatives from the selected chemical library, we adopted a computational ligand screening protocol to rapidly identify potential SIRT1 activators. For this purpose, we coupled a molecular docking-based investigation with the evaluation of relative ligand binding energy, employing the experimentally solved structure of the SIRT1 enzyme in complex with resveratrol as described [12,43]. As mentioned, we employed an in-house chemical library composed of compounds designed as resveratrol-like molecules. Because of their chemical structures, we assumed that these molecules might share a comparable mechanism described for resveratrol regarding SIRT1 activation. Figure 3A shows the mechanism of action of resveratrol regarding the activation of the SIRT1 enzyme. The natural compound is able to target a particular region of the protein situated in the N-terminal domain (NTD). Particularly, resveratrol can interact with the NTD by targeting three different active sites (#site1, #site2, and #site3 in Figure 3A), as established by in vitro experiments [35].

The mentioned binding sites differently participate in activating the SIRT1 enzyme. In effect, mutagenesis analyses suggested that the interactions with #site1 and #site2 are crucial for SIRT1 activation, but #site3 is defined as an “accessory site”, demonstrating a smaller influence on the activation of SIRT1. Consequently, two resveratrol molecules enable the interaction with the AMC-peptide and the SIRT1-NTD and are exceptionally important in promoting a powerful binding between the peptide and SIRT1, increasing the activity of the enzyme. Considering the described mechanism of action, we established a molecular docking protocol capable of evaluating the binding affinity of a compound into the three different active sites of SIRT1. Therefore, we used our chemical library in this computational step, assessing the affinity of the derivatives for these binding sites. To select the most likely compounds, we considered only those compounds demonstrating a satisfactory affinity for each site comparable or better with respect to the values observed for resveratrol (Table 2). This evaluation was accomplished considering the docking score calculated by Glide software (Glide release 2018, Schrödinger LLC, New York, 2018), using the scoring function extra precision (XP) and performing a rescoring step using the scoring function standard precision (SP), coupled with the evaluation of relative ligand binding energy (ΔG_bind_), evaluated by Prime software adopting the MM/GBSA technique (Prime release 2018, Schrödinger LLC, New York, NY, USA, 2018). The additional step of the developed in silico protocol involved the evaluation of the drug-like profile of molecules showing satisfactory GlideScore values. To this end, we submitted the selected derivatives to the QikProp application (QikProp release 2018, Schrödinger LLC, New York, NY, USA, 2018) and the FAFDrugs4 webserver. Following this calculation, we chose only those derivatives showing satisfactory physicochemical properties considering the output found for the reference compound (resveratrol). Accordingly, as stated in Table 2, the selected molecules presented satisfactory parameters such as LogP, solubility (LogS), and capability to cross biological membranes. Additionally, the selected SIRT1 activators were predicted to be devoid of cardiotoxicity. The evaluation of oral absorption indicated a favorable expected profile for the selected derivatives with respect to resveratrol. Moreover, the output of FAFDrugs4 indicated that the selected molecules are not pan-assay interference compounds (PAINS), even if the output underlined a risk warning for derivatives having catechol and phenol moiety. Finally, the in silico strategy identified six possible SIRT1 activators with satisfactory drug-like profiles (Table 2).

Employing the described computational approach, we selected six derivatives showing computational scores comparable or even better with respect to those found for resveratrol. Figure 3 describes the docking output of compound **3d**, representing one of the best-performing derivatives. This molecule can strongly interact with all three binding sites on SIRT1 as suggested by GlideScores and ΔG_bind_ values. Compound **3d** within #site 1 is able to H-bind the sidechains of E230, T209, and the p53-AMC-peptide, also forming a relevant network of hydrophobic interactions with the residues in the binding site and the p53-AMC-peptide, including π-π stacking with the aromatic moiety of the mentioned peptide. Considering #site2, **3d** can target the sidechain of Q222, E214, D298, and the p53-AMC-peptide by forming several H-bonds. Hydrophobic contacts were detected with the aromatic moiety of the p53-AMC-peptide. Finally, when **3d** was docked into #site3, it was able to target D292 and D298 by two H-bonds and, in addition, we observed an H-bond with the residue K444, similar to the reference molecule resveratrol. According to the computational output, **3d** is potentially established as one of the best-performing SIRT1 activators. After that, to validate the in silico strategy, the possible SIRT1 activators selected by our computational protocol were submitted to the biological evaluation.

### 2.3. In Vitro SIRT1 Activity

**Figure 4:**
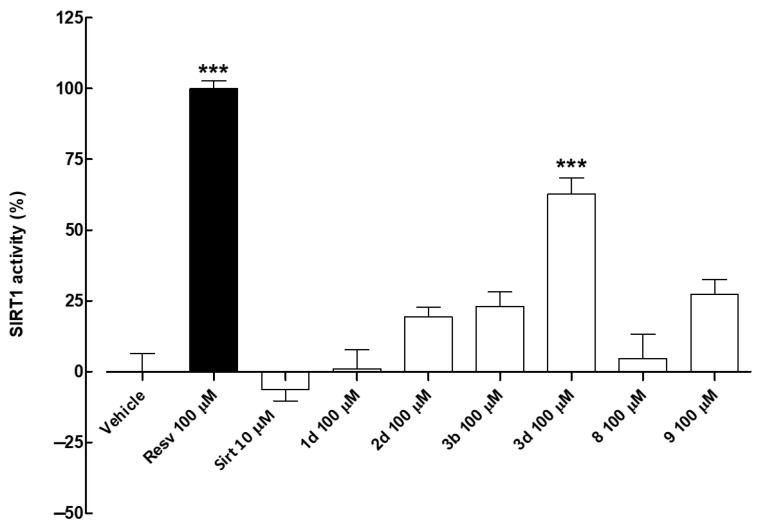
Effects of the screened compounds on isolated SIRT1 enzyme activity. SIRT1 activity is expressed as % of the effect promoted by the reference compound resveratrol (Resv) 100 µM. The sirtuin inhibitor sirtinol (Sirt) 10 µM was used as a negative control. Results are shown as mean ± SEM. * indicates significant difference vs. vehicle (*** *p* < 0.001).

In the cell-free assay, resveratrol (Resv, 100 µM) was used as a reference activator (positive control) [12,43,50] while sirtinol (Sirt, 10 µM) as a known sirtuin inhibitor (negative control). The effects of the screened compounds on isolated SIRT1 enzyme activity were evaluated as a percentage of SIRT1 activation. Results showed that compounds **1d** (R = 2-OH, R_2_, R_4_ = OH) and **8** (R = 3-Cl, R_2_, R_4_ = OH) (both 100 µM) had no effect on SIRT1 activity (Figure 4 and Table 3). Conversely, compounds **2d** (R = 3-OH, R_2_, R_4_ = OH), **3b** (R = 4-OH, R_2_ = OH), **3d** (R = 4-OH, R_2_, R_4_ = OH), and **9** (R = Cl, R_2_, R_4_ = OH), at the tested concentration of 100 µM, markedly improved SIRT1 activity compared with the vehicle. Among the tested compounds, **3d** was the most effective activator of the isolated SIRT1 enzyme (% of SIRT1 activity compared with resveratrol 100 µM: 62.8 ± 5.6). Therefore, compound **3d** was selected for further investigation.

**Figure 5:**
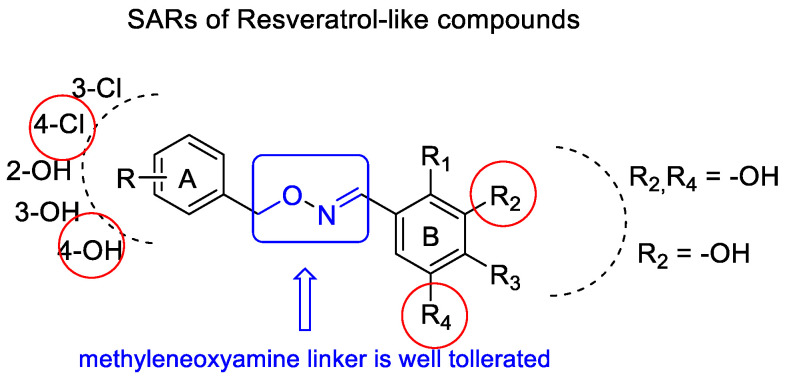
Graphic representation of SARs resveratrol-like compounds.

SAR analysis highlighted that the methyleneoxyamine linker between the two aromatic portions is well tolerated by the SIRT1 enzyme and that the hydroxy substitutions on rings A and B in the same position as that of resveratrol, or at least a hydroxyl group in the 3-position of ring B, are necessary to achieve the optimal activity (Figure 5).

**Figure 6:**
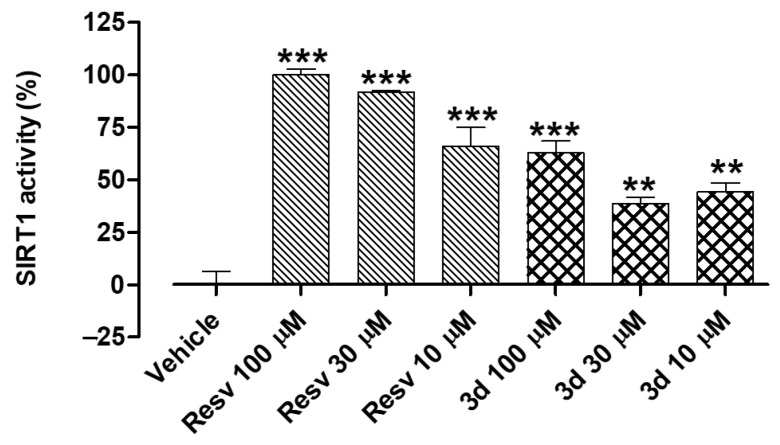
Effects of increasing concentrations of resveratrol and the synthetic derivative **3d** (10, 30, and 100 µM) on the isolated SIRT1 enzyme. SIRT1 activity is expressed as % of the effect promoted by the reference compound resveratrol (Resv) 100 µM. Results are shown as mean ± SEM. * indicates significant difference vs. vehicle (** *p* < 0.01; *** *p* < 0.001).

To perform an in-depth mechanistic investigation of compound **3d**, the resveratrol derivative was tested at three different concentrations (10, 30, and 100 µM) in the cell-free assay (Figure 6). In this set of experiments, both compound **3d** and the reference compound resveratrol (Resv) exhibited an almost concentration–response effect. This result confirmed the SIRT1-activating properties of compound **3d** and represented a rationale for studying its potential preventive effects in cultured endothelial cells.

### 2.4. Preventive Effects of Resveratrol and Compound ***3d*** on Cell Viability in H_2_O_2_-Challenged HUVECs

**Figure 7:**
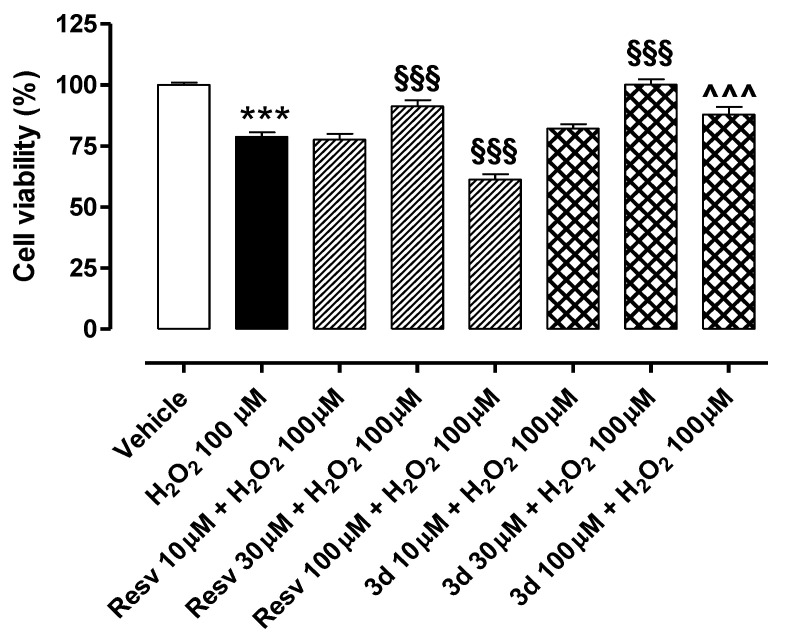
Preventive effects of resveratrol and compound **3d** against H_2_O_2_-induced reduction in cell viability: A perfect example of a biphasic concentration–response curve. Bars indicate cell viability (%) of HUVECs exposed to H_2_O_2_ 100 µM for 2 h. Pre-incubation of resveratrol and compound **3d** (both 30 µM) for 1 h significantly prevented H_2_O_2_-induced reduction in cell viability. Conversely, both resveratrol and compound **3d** 10 µM and 100 µM did not preserve cell viability in HUVECs exposed to the pro-oxidative stimulus. Data are shown as mean ± SEM. * indicates significant difference vs. vehicle (*** *p* < 0.001), § indicates significant difference vs. H_2_O_2_ 100 µM (§§§ *p* < 0.001) and ^ indicates significant difference vs. Resv 100 µM (^^^ *p* < 0.001).

The exposure of HUVECs to H_2_O_2_ 100 µM for 2 h led to a significant reduction in cell viability (% of cell viability vs. vehicle: 78.8 ± 1.8) (Figure 7). Both resveratrol (Resv 10, 30, and 100 µM) and compound **3d** (10, 30, and 100 µM), pre-incubated for 1 h before the pro-oxidative stimulus, exhibited a singular biphasic effect. Indeed, they significantly preserved cell viability at a concentration of 30 µM, with the synthetic derivative being slightly more effective than the natural polyphenol (% of cell viability vs. vehicle: 91.3 ± 2.5 for Resv 30 µM; 100.2 ± 2.2 for compound **3d** 30 µM). Conversely, both lower (10 µM) and higher (100 µM) concentrations did not produce protective effects on HUVECs exposed to H_2_O_2_ 100 µM. Indeed, cell viability vs. vehicle was 76.0 ± 2.4% for Resv 10 µM and 82.1 ± 1.9% for **3d** 10 µM. Notably, the cell viability of endothelial cells treated with resveratrol 100 µM was significantly lower than that observed in cells treated only with H_2_O_2_ (% of cell viability vs. vehicle for Resv 100 µM: 61.3 ± 2.1). Such toxicity was not measured in cells treated with compound **3d** 100 µM (% of cell viability vs. vehicle for **3d** 100 µM: 87.9 ± 3.2), but the effect observed at 100 µM was lower than that promoted at 30 µM.

### 2.5. Potential Role of Sirtuins in the Preventive Effects of Resveratrol and Compound ***3d*** on Cell Viability in HUVECs Exposed to H_2_O_2_

**Figure 8:**
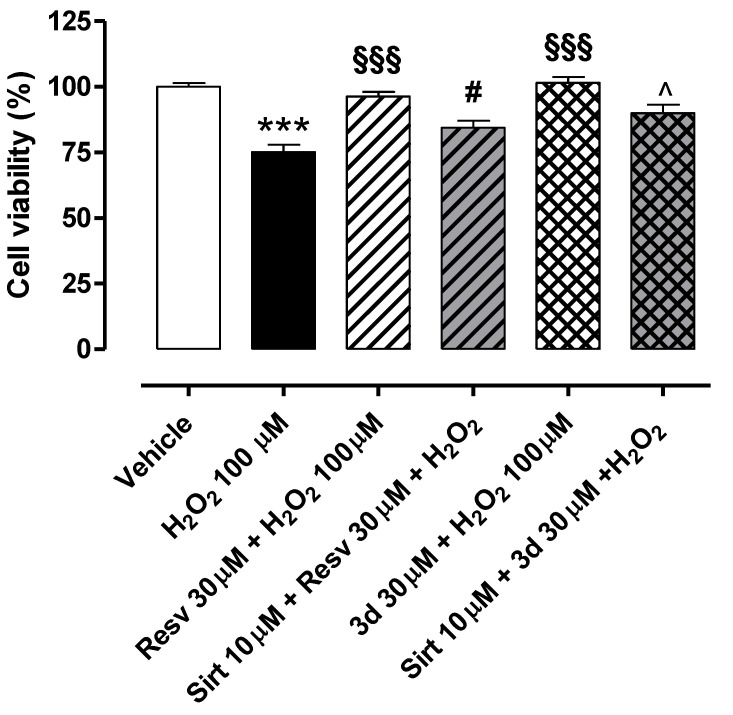
Preventive effects of resveratrol and compound **3d** against H_2_O_2_-induced reduction in cell viability: Potential role of sirtuins. Bars indicate cell viability (%) of HUVECs treated with H_2_O_2_ 100 µM for 2 h. In the absence of the sirtuin inhibitor sirtinol (Sirt 10 µM), both resveratrol (Resv) and compound **3d** (30 µM), incubated for 1 h, significantly protected endothelial cells from pro-oxidative cell damage. Conversely, in the presence of Sirt 10 µM, the preventive effects of resveratrol and compound **3d** against H_2_O_2_-induced reduction in cell viability were significantly reduced. Data are shown as mean ± SEM. * indicates significant difference vs. vehicle (*** *p* < 0.001), § indicates significant difference vs. H_2_O_2_ 100 µM (§§§ *p* < 0.001), # indicates significant difference vs. Resv 30 µM + H_2_O_2_ 100 µM (# *p* < 0.05) while ^ indicates significant difference vs. **3d** 30 µM + H_2_O_2_ 100 µM (^ *p* < 0.05).

In a separate set of experiments aimed at evaluating the potential role of sirtuins in the preventive effects of the SIRT1 activators resveratrol and compound **3d** (Figure 8), the most effective concentration of both compounds (30 µM) was tested in the presence and absence of the sirtuin inhibitor sirtinol (Sirt 10 µM). The exposure of HUVECs to H_2_O_2_ 100 µM for 2 h led to a significant decrease in cell viability (% of cell viability vs. vehicle: 75.2 ± 2.8). In the presence of sirtinol, the preventive effects exhibited by resveratrol and compound **3d** against the cell viability decrease were significantly reduced, confirming the possible involvement of sirtuins in their cytoprotective effects (% of cell viability vs. vehicle for **3d** 30 µM in the absence of Sirt 10 µM: 101.6 ± 2.2; % of cell viability vs. vehicle for **3d** 30 µM in the presence of Sirt 10 µM: 90.0 ± 3.3; % of cell viability vs. vehicle for Resv 30 µM in the absence of Sirt 10 µM: 96.3 ± 1.8; % of cell viability vs. vehicle for **3d** 30 µM in the presence of Sirt 10 µM: 84.5 ± 2.7).

### 2.6. Preventive Effects of Resveratrol and Compound ***3d*** against H_2_O_2_-Induced Intracellular ROS Production

**Figure 9:**
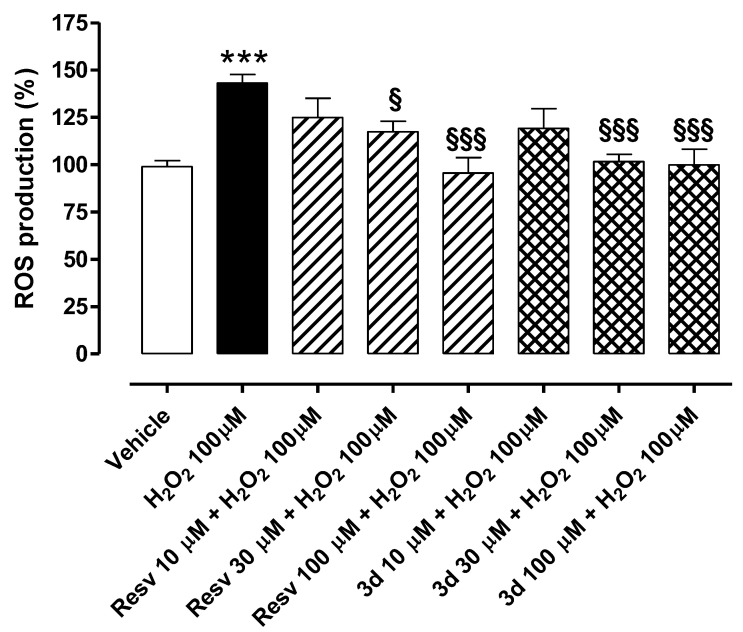
Preventive effects of resveratrol and **3d** against H_2_O_2_-induced intracellular ROS production in HUVECs. Bars indicate intracellular ROS production (%) in HUVECs exposed to H_2_O_2_ 100 µM for 2 h. Pre-incubation of resveratrol (Resv) and **3d** (10, 30, and 100 µM) for 1 h prevented H_2_O_2_-induced ROS production in a concentration-dependent manner. Data are shown as mean ± SEM. * indicates significant difference vs. vehicle (*** *p* < 0.001) while § indicates significant difference vs. H_2_O_2_ 100 µM (§ *p* < 0.05; §§§ *p* < 0.001).

Treatment of HUVECs with H_2_O_2_ 100 µM for 2 h significantly enhanced ROS levels (% of ROS vs. vehicle: 143.1 ± 4.5) (Figure 9). Pre-incubation of resveratrol (Resv 10, 30, and 100 µM) before administration of the pro-oxidative stimulus prevented such an increase (% of ROS vs. vehicle: 95.6 ± 8.2 for Resv 100 µM; 117.4 ± 5.4 for Resv 30 µM; 124.9 ± 10.1 for Resv 10 µM). Furthermore, pre-treatment of HUVECs with the synthetic derivative compound **3d** reduced intracellular ROS production following exposure to H_2_O_2_ 100 µM in a concentration-dependent manner (% of ROS vs. vehicle: 99.8 ± 8.3 for **3d** 100 µM; 101.7 ± 3.8 for **3d** 30 µM; 119.1 ± 10.6 for **3d** 10 µM). The effects were statistically significant at concentrations of 30 and 100 µM for both compounds. Noteworthily, compound **3d** maintained the antioxidant properties of resveratrol and seemed even more effective than the reference compound at a concentration of 30 µM.

### 2.7. Potential Role of Sirtuins in the Preventive Effects of Resveratrol and Compound ***3d*** against H_2_O_2_-Induced Intracellular ROS Production

**Figure 10:**
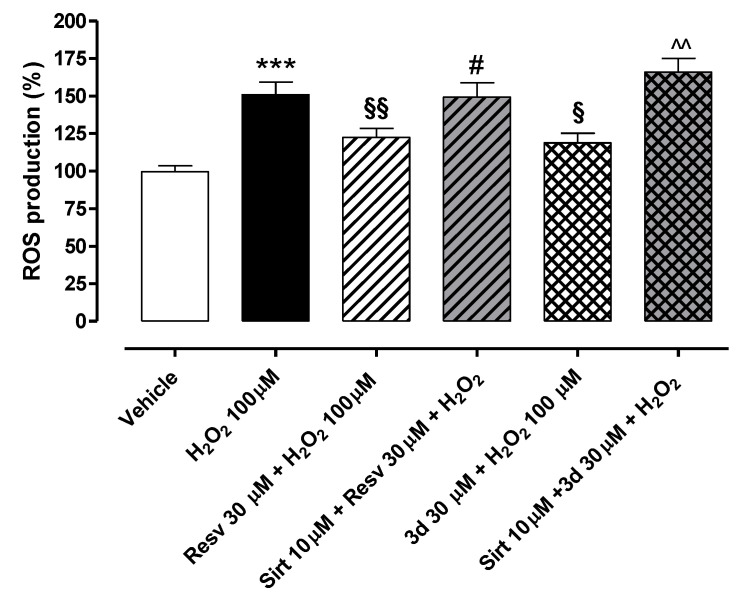
Preventive effects of resveratrol and compound **3d** against H_2_O_2_-induced increase in intracellular ROS production: Potential role of sirtuins. Bars indicate intracellular ROS production (%) in HUVECs treated with H_2_O_2_ 100 µM for 2 h. In the absence of the sirtuin inhibitor sirtinol (Sirt 10 µM), both resveratrol (Resv) and **3d** (30 µM) incubated for 1 h significantly protected endothelial cells from the ROS increase. Conversely, in the presence of Sirt 10 µM, the preventive effects of resveratrol and **3d** against H_2_O_2_-induced increase in ROS were almost abolished. Data are shown as mean ± SEM. * indicates significant difference vs. vehicle (*** *p* < 0.001), § indicates significant difference vs. H_2_O_2_ 100 µM (§ *p* < 0.05; §§ *p* < 0.01), # indicates significant difference vs. Resv 30 µM + H_2_O_2_ 100 µM (# *p* < 0.05) while ^ indicates significant difference vs. **3d** 30 µM + H_2_O_2_ 100 µM (^^ *p* < 0.01).

In another set of experiments, resveratrol and compound **3d** (30 µM both) were tested in the presence and absence of the sirtuin inhibitor sirtinol (Sirt 10 µM) to further investigate the possible involvement of sirtuins in the protective effects exhibited by both compounds in endothelial cells injured by H_2_O_2_ (Figure 10). Concentrations of resveratrol and compound **3d** were selected on the basis of the previous cell viability assays, where they exhibited potential toxicity at high concentrations (100 µM). Treatment of HUVECs with H_2_O_2_ 100 µM for 2 h significantly enhanced intracellular ROS production (% of ROS vs. vehicle: 150.8 ± 8.5). Following the pre-incubation of sirtinol, the preventive effects promoted by resveratrol and compound **3d** against intracellular ROS production were almost abolished, thus suggesting the potential role of sirtuins in the antioxidant effects promoted by the two SIRT1 activators (% of ROS vs. vehicle for **3d** 30 µM in the absence of Sirt 10 µM: 118.8 ± 6.4; % of ROS production vs. vehicle for **3d** 30 µM in the presence of Sirt 10 µM: 165.8 ± 9.3; % of ROS production vs. vehicle for Resv 30 µM in the absence of Sirt 10 µM: 122.4 ± 6.0; % of ROS production vs. vehicle for **3d** 30 µM in the presence of Sirt 10 µM: 149.1 ± 10.0).

## 3. Discussion

Resveratrol, a polyphenol particularly abundant in red grapes, is considered an effective SIRT1 activator, but it is characterized by very low bioavailability that limits its clinical use. In recent years, the chemical structure of resveratrol has been extensively modified to synthesize derivatives with both an improved pharmacokinetic profile and preserved pharmacological properties in the vascular endothelium. In this context, starting from the resveratrol chemical structure and with the aim of identifying new SIRT1 activators, we used an in-house library of synthetic small resveratrol-like molecules. We first applied a computational screening approach using the crystal structure of the SIRT1 enzyme in complex with resveratrol coupled with the evaluation of relative ligand binding energy to rapidly identify the most promising SIRT1 activators within the library.

Based on the results of the in silico approach, the potential SIRT1-activating properties of the six selected compounds were then evaluated on the isolated SIRT1 enzyme. Indeed, SIRT1 plays a pivotal role in the maintenance of vascular homeostasis and represents a major pharmacological target of the natural compound resveratrol [35,36,37,38]. We hypothesized that our library of synthetic derivatives of resveratrol might also effectively activate the isolated SIRT1 enzyme. Results showed that compound **3d** is the most effective activator of the SIRT1 enzyme, although weaker than resveratrol. This evidence indicates that modest changes in the chemical structure of resveratrol can markedly affect the interaction of the natural compound with the SIRT1 enzyme.

Oxidative stress is a major trigger for endothelial dysfunction, vascular damage, and related CVDs [1,2,3]. Therefore, the discovery of novel compounds able to prevent oxidative stress in the endothelium represents a compelling issue. The activation of SIRT1 is a major mechanism of action responsible for the antioxidant effects of resveratrol in the vascular endothelium [51,52]. Indeed, SIRT1 is an epigenetic regulator of the endogenous antioxidant defense system, which promotes the expression of antioxidant genes (i.e., SOD, CAT, GPx) through activation of the Nrf2/ARE pathway [30]. Given the promising SIRT1-activating properties exhibited in the cell-free assay, compound **3d** underwent further pharmacological investigation in human endothelial cells exposed to an oxidative stimulus. In particular, we reproduced the oxidative environment, typical of endothelial dysfunction, by means of the sustained treatment of human endothelial cells with micromolar concentrations of H_2_O_2_, as previously reported [29]. In this set of experiments, both resveratrol and compound **3d** significantly preserved cell viability. Of note, they showed a biphasic concentration–response curve, being toxic or ineffective at high concentrations (100 µM) and cytoprotective at lower concentrations, as previously demonstrated for the natural polyphenol [53,54].

To corroborate the preliminary results on the cytoprotective effects of resveratrol and compound **3d** against vascular oxidative damage, we then measured the intracellular levels of ROS. Indeed, the exposure of endothelial cells to oxidative stimuli not only reduces cell viability but also leads to increased ROS production [28]. In this work, we demonstrated that resveratrol and compound **3d** significantly and almost concentration-dependently protect endothelial cells from H_2_O_2_-induced intracellular ROS increase, as already reported for the natural SIRT1 activator tested in a similar concentration range [55].

Of note, in the presence of the sirtuin inhibitor sirtinol, the preventive effects of resveratrol and compound **3d** against H_2_O_2_-induced cell viability reduction and ROS production were partially reduced. This evidence, which strengthens the results obtained in the cell-free assay, finds partial confirmation in the current literature. Indeed, Pan and colleagues observed that, in the presence of the sirtuin inhibitor Ex527, the preventive effects of resveratrol on the cell viability of HUVECs exposed to oxidative stimuli were significantly decreased [51], further indicating the potential role of SIRT1 in the vascular properties of both resveratrol and, reasonably, its SIRT1-activating derivatives. Other authors demonstrated that resveratrol reduces endothelial oxidative stress by increasing both the activity of SOD and glutathione levels [56], likely via activation of SIRT1 [51]. Hence, our data confirm that compound **3d**, despite the changes in the chemical structure of resveratrol, can still activate SIRT1 and promote beneficial effects against oxidative stress in the vascular endothelium.

**Figure 11:**
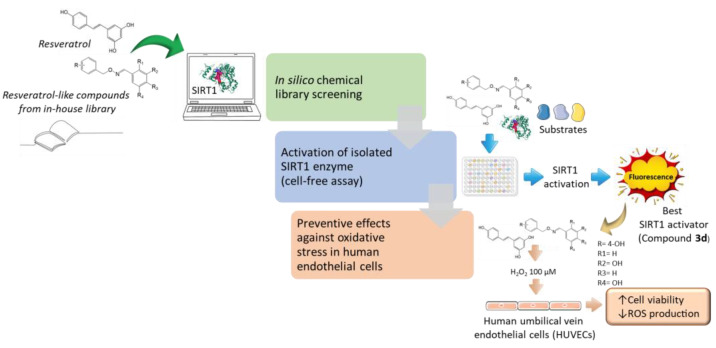
Summary of the work. Resveratrol-like compounds from our in-house library were first screened in silico to predict their ability to activate SIRT1 enzyme and bioavailability. Then, six selected compounds were tested in vitro to assess the potential activation of isolated SIRT1 enzyme. The best SIRT1 activator (compound **3d**) underwent pharmacological investigation in human endothelial cells (HUVECs). In this final set of experiments, both the reference SIRT1 activator resveratrol and compound **3d** prevented H_2_O_2_-induced cell viability reduction and ROS production, likely via activation of SIRT1.

The workflow of our research steps is depicted in Figure 11.

Of course, as sirtinol is a non-selective SIRT1 inhibitor, we cannot completely exclude the potential role of different sirtuin isoforms in the pharmacological effects of compound **3d** and resveratrol. Indeed, resveratrol has also been described as an activator of SIRT2 [57], as well as a potent modulator of SIRT1-5 pathways, which are reported to be responsible for its antioxidant effects in endothelial cells [58]. However, in this work, we focused on SIRT1 as it is one of the most characterized sirtuin isoforms in the cardiovascular system and is considered a novel target for the prevention of vascular oxidative stress, inflammation, and ageing [29,59,60]. Moreover, modulation of the SIRT1 enzyme seems to be responsible for the pharmacological effects of resveratrol in non-vascular cells, such as tenocytes [61], chondrocytes [62], fibroblasts [63], and cancer cells [64]. Therefore, our results could represent a starting point for the potential clinical use of SIRT1-activating resveratrol-like compounds not only in the prevention of vascular oxidative stress but also in the treatment of a plethora of diseases associated with the SIRT1-mediated loss of cell function and impaired tissue homeostasis.

## 4. Materials and Methods

### 4.1. Chemistry

All analytical-grade reagents and solvents were purchased from Merck KGaA (Darmstadt, Germany) and were used without further purification. ^1^H and ^13^C NMR spectra were collected at 25 °C using a Bruker Ultrashield^TM^ 400 MHz (Switzerland). The following abbreviations are used: Singlet (s), doublet (d), triplet (t) doublet of doublets (dd), and multiplet (m). Melting points (m.p.) were measured with a Leica Galen III microscope (Leica/Cambridge Instruments) and were uncorrected. Reactions were monitored by thin-layer chromatography (TLC) on silica gel plates containing a fluorescent indicator (Merck Silica Gel 60 F254); spots were detected under UV light (254 nm). Evaporation was carried out under reduced pressure using a rotating evaporator, and Na_2_SO_4_ was always used as the drying agent. Mass spectra (ESI-MS) were recorded with a high-resolution Q Exactive plus Orbitrap spectrometer (Thermo Fisher Scientific, Waltham, MA, USA), with a resolution of 140,000 at *m*/*z* 200.

#### 4.1.1. General Procedure for the Synthesis of Hydroxymethylphenols **13–15**

To an ethanolic solution of the opportune, commercially available hydroxybenzaldehyde **10–12** (10.15 mmol) at 0 °C, NaBH_4_ (5.07 mmol) was added at a ratio of 2:1. The reaction mixture was stirred at room temperature until the starting material disappeared (TLC analysis). The mixture was evaporated to dryness and the resulting crude was added with HCl 1N. The water solution was extracted with Et_2_O and the organic phase was washed (3×) with water, dried (Na_2_SO_4_), filtered, and evaporated under reduced pressure to obtain a crude solid.

#### 4.1.2. 2-(Hydroxymethyl)phenol, **13**

Compound **13** was obtained starting from 2-hydroxybenzaldehyde **10**, following the general procedure. The crude was purified by trituration with Et_2_O/hexane affording compound **13** as a white solid. Yield 86%. M.p.: 49–52 °C. ^1^H-NMR (400 MHz; CDCl_3_) δ: 7.24–7.19 (m, 1H, Ar); 7.06–7.02 (m, 1H, Ar); 6.90–6.84 (m, 2H, Ar); 4.87 (s, 2H, -CH_2_O); 2.21 (bs, 1H, -OH); 1.68 (bs, 1H, -OH).

#### 4.1.3. 3-(Hydroxymethyl)phenol, **14**

Compound **14** was obtained starting from the 3-hydroxybenzaldehyde **11**, following the general procedure. The crude was purified by trituration with hexane affording compound **14** as a white solid. Yield 86%. M.p.: 53–55 °C. ^1^H-NMR (400 MHz; DMSO-*d_6_*) δ: 9.26 (s, 1H, -OH phenol); 7.08 (t, 1H, *J* = 8.00 Hz, Ar); 6.73–6.69 (m, 2H, Ar); 6.61–6.58 (m, 1H, Ar); 5.08 (t, 1H, *J* = 5.6 Hz, -OH); 4.40 (d, 2H, *J* = 5.6 Hz, -CH_2_O).

#### 4.1.4. 4-(Hydroxymethyl)phenol, **15**

Compound **15** was obtained starting from the 4-hydroxybenzaldehyde **12**, following the general procedure. The crude was purified by trituration with hexane affording compound **15** as a white solid. Yield 88%. M.p.: 72–75 °C. ^1^H-NMR (400 MHz; DMSO-*d_6_*) δ: 9.23 (s, 1H, -OH phenol); 7.10–7.08 (m, 2H, Ar); 6.70–6.68 (m, 2H, Ar); 4.34 (s, 2H, -CH_2_O).

#### 4.1.5. General Procedure for the Synthesis of 2-Hydroxybenzyl-oxy-isoindolin-1,3-dione **16–18**

To a stirred solution, the appropriate hydroxymethyl)phenol **13–15** (8.05 mmol) and anhydrous THF (8.6 mL) were added under argon Triphenylphosphine, *N*-hydroxyphthalimide, and DEAD with a ratio of 1:1.1:1.1:1.1 [49]. The reaction mixture was stirred at room temperature for 6 h TLC (analysis) and then evaporated to dryness. The crude products were purified by flash chromatography on a silica gel column.

#### 4.1.6. 2-((2-Hydroxybenzyl)-oxy)-isoindolin-1,3-dione, **16**

Compound **16** was purified by eluting the column with a mixture of CHCl_3_/EtOAc/hexane at a ratio of 3:3:4. White solid, 22% yield, m.p.: 169–172 °C; ^1^H NMR (400 MHz, CDCl_3_) δ: 7.90–7.86 (m, 2H, Ar); 7.81–7.76 (m, 2H, Ar); 7.63 (bs, 1H, -OH phenol); 7.35–7.31 (m, 1H, Ar); 7.25–7.23 (m, 1H, Ar); 7.05–7.03 (m, 1H, Ar); 6.93–6.88 (m, 1H, Ar); 5.25 (s, 2H, -CH_2_O).

#### 4.1.7. 3-((2-Hydroxybenzyl)-oxy)-isoindolin-1,3-dione, **17**

Compound **17** was purified by eluting the column with a mixture of hexane/EtOAc at a ratio of 6.5:3.5. White solid, 61% yield, m.p.: 155–158 °C; ^1^H NMR (400 MHz, DMSO-*d_6_*) δ: ^1^H NMR (400 MHz, DMSO-*d_6_*) δ: 9.55 (s, 1H, -OH); 7.86 (s, 4H, Ar); 7.18 (t, 1H, *J* = 7.6 Hz, Ar); 6.92–6.88 (m, 2H, Ar); 6.78–6.76 (m, 1H, Ar); 5.06 (s, 2H, -CH_2_O).

#### 4.1.8. 4-((2-Hydroxybenzyl)-oxy)-isoindolin-1,3-dione, **18**

Compound **18** was purified by eluting the column with a mixture of hexane/EtOAc at a ratio of 7:3. White solid, 35% yield, m.p.: 145–148 °C; ^1^H NMR (400 MHz, DMSO-*d_6_*) δ: 9.64 (bs, 1H, -OH); 7.84 (s, 4H, Ar); 7.30–7.28 (m, 2H, Ar); 6.76–6.74 (m, 2H, Ar); 5.02 (s, 2H, -CH_2_O).

#### 4.1.9. General Procedure for the Synthesis of Aminooxymethyl-Phenols Hydrochlorides **19–21**

To a solution of the appropriate 2-hydroxybenzyl-oxy-isoindolin-1,3-dione, **16–18** (0.74 mmol) was added to ammonia in a methanol solution (7 M) at a ratio of 1:182. The reaction mixture was stirred at room temperature until the disappearance of the starting compound (TLC monitoring). After evaporation to dryness, the solid residue was dissolved in Et_2_O and treated, at 0 °C, with an excess of Et_2_O·HCl (pH 3–4) to yield a solid precipitate, which was filtered and triturated with Et_2_O to give pure **19–21**.

#### 4.1.10. 2-((Aminooxy) methyl)-phenol hydrochloride, **19**

Compound **19** was obtained as a white solid, 78% yield, m.p.: 104–107 °C; ^1^H NMR (400 MHz, DMSO-*d_6_*) δ: 10.72 (bs, 3H, -NH_3_); 7.25–7.19 (m, 2H, Ar); 6.92–6.90 (m, 1H, Ar); 6.83–6.79 (m, 1H, Ar); 4.99 (s, 2H, -CH_2_O).

#### 4.1.11. 3-((Aminooxy) methyl)-phenol hydrochloride, **20**

Compound **20** was obtained as a white solid, 73% yield, m.p.: 128–130 °C; ^1^H NMR (400 MHz, DMSO-*d_6_*) δ: ^1^H NMR (400 MHz, DMSO-*d_6_*) δ: 10.72 (bs, 3H, -NH_3_); 9.65 (bs, 1H, -OH); 7.21–7.17 (m, 1H, Ar); 6.81–6.78 (m, 3H, Ar); 4.93 (s, 2H, -CH_2_O).

#### 4.1.12. 4-((Aminooxy) methyl)-phenol hydrochloride, **21**

Compound **21** was obtained as a white solid, 54% yield, m.p.: 104–106 °C; ^1^H NMR (400 MHz, DMSO-*d_6_*) δ: 10.85 (bs, 3H, -NH_3_); 9.73 (bs, 1H, -OH); 7.22–7.19 (m, 2H, Ar); 6.79–6.77 (m, 2H, Ar); 4.86 (s, 2H, -CH_2_O).

#### 4.1.13. General Procedure for the Synthesis of (E)-mono and Polyhydroxy Substituted benzaldehyde O-(Benzyl) oximes **1a–e**, **2a–e**, **3b**, **3d–e**

To a solution of the commercially available hydroxybenzaldehyde **22–26** (0.285 mmol) in MeOH (4 mL), an aqueous solution (1 mL) of the opportune hydroxylamine hydrochloride **19–21** (0.285 mmol) was added [65]. The reaction mixture was stirred at room temperature until the disappearance of the starting material (TLC analysis). The mixture was evaporated to dryness and the resulting crude was added to water and extracted with EtOAc. The organic phase was washed (3×) with water, dried (Na_2_SO_4_), filtered, and evaporated under reduced pressure to obtain a crude solid. The *E* configuration was obtained [48,66].

#### 4.1.14. (E)-2-Hydroxy-benzaldehyde-O-(2-hydroxybenzyl)-oxime **1a**

The crude was purified by trituration with hexane affording compound **1a** a white solid, 71% yield, m.p.: 102–104 °C; ^1^H NMR (400 MHz, DMSO-*d_6_*) δ: 9.92 (bs, 1H, -OH); 9.63 (bs, 1H, -OH); 8.40 (s, 1H, N=CH); 7.49–7.47 (m, 1H, Ar); 7.28–7.22 (m, 2H, Ar); 7.16–7.11 (m, 1H, Ar); 6.89–6.83 (m, 3H, Ar); 6.81–6.76 (m, 1H, Ar); 5.12 (s, 2H, -CH_2_O). ^13^C-NMR (100 MHz; DMSO-*d_6_*) δ: 156.6, 156.0, 147.7, 131.7, 130.9, 129.7, 128.3, 123.0, 119.8, 119.3, 118.0, 116.6, 115.7, 71.3. *m*/*z* ESI-MS: [M + H]^+^ 244.09.

#### 4.1.15. (E)-3-Hydroxy-benzaldehyde-O-(2-hydroxybenzyl)-oxime **1b**

The crude was purified by trituration with hexane affording compound **1b** a white solid, 72% yield, m.p.: 112–114 °C; ^1^H NMR (400 MHz, DMSO-*d_6_*) δ: 9.58 (s, 1H, -OH); 9.57 (s, 1H, -OH); 8.17 (s, 1H, N=CH); 7.26–7.24 (m, 1H, Ar); 7.22–7.18 (m, 1H, Ar); 7.13–7.11 (m, 1H, Ar); 7.03–6.99 (m, 2H, Ar) 6.84–6.82 (m, 1H, Ar); 6.81–6.77 (m, 2H, Ar); 5.11 (s, 2H, -CH_2_O). ^13^C-NMR (100 MHz; MeOD) δ: 158.8, 156.7, 150.1, 134.9, 131.3, 130.8, 130.2, 125.2, 120.4, 119.9, 118.1, 116.2, 114.0, 72.6. *m*/*z* ESI-MS: [M + H]^+^ 244.09.

#### 4.1.16. (E)-4-Hydroxy-benzaldehyde-O-(2-hydroxybenzyl)-oxime **1c**

The crude was purified by trituration with hexane affording compound **1c** as a white solid, 62% yield, m.p.: 119–121 °C; ^1^H NMR (400 MHz, DMSO-*d_6_*) δ: 9.87 (s, 1H, -OH); 9.56 (s, 1H, -OH); 8.14 (s, 1H, N=CH); 7.43–7.41 (m, 2H, Ar); 7.26–7.24 (m, 1H, Ar); 7.14–7.09 (m, 1H, Ar); 6.84–6.81 (m, 2H, Ar); 6.79–6.76 (m, 2H, Ar); 5.07 (s, 2H, -CH_2_O). ^13^C-NMR (100 MHz; MeOD) δ: 159.2, 155.3, 148.8, 129.9, 128.8, 128.3, 124.0, 123.4, 119.0, 115.2, 114.9, 71.0. *m*/*z* ESI-MS: [M + H]^+^ 244.09.

#### 4.1.17. (E)-3,5-Dihydroxy-benzaldehyde-O-(2-hydroxybenzyl)-oxime **1d**

The crude was purified by trituration with hexane affording compound **1d** as a white solid, 51% yield, m.p.: 132–134 °C; ^1^H NMR (400 MHz, DMSO-*d_6_*) δ: 9.56 (s, 1H, -OH); 9.40 (s, 2H, -OH); 8.06 (s, 1H, N=CH); 7.25–7.23 (m, 1H, Ar); 7.15–7.10 (m, 1H, Ar); 6.84–6.82 (m, 1H, Ar); 6.79–6.76 (m, 1H, Ar); 6.46 (d, 2H, *J* = 2.4 Hz, Ar); 6.22 (t, 1H, *J* = 2.4 Hz, Ar); 5.08 (s, 2H, -CH_2_O). ^13^C-NMR (100 MHz; MeOD) δ: 158.5, 155.3, 148.9, 133.5, 129.9, 128.8, 123.8, 119.0, 114.8, 105.1, 103.9, 71.2. *m*/*z* ESI-MS: [M + H]^+^ 260.09.

#### 4.1.18. (E)-2,4-Dihydroxy-benzaldehyde-O-(2-hydroxybenzyl)-oxime **1e**

The crude was purified by trituration with hexane affording compound **1e** as a white solid, 59% yield, m.p.: 143–145 °C; ^1^H NMR (400 MHz, DMSO-*d_6_*) δ: 9.90 (bs, 1H, -OH); 9.81 (bs, 1H, -OH); 9.59 (bs, 1H, -OH); 8.27 (s, 1H, N=CH); 7.27–7.23 (m, 2H, Ar); 7.15–7.11 (m, 1H, Ar); 6.85–6.82 (m, 1H, Ar); 6.79–6.76 (m, 1H, Ar); 6.29–6.27 (m, 2H, Ar); 5.05 (s, 2H, -CH_2_O). ^13^C-NMR (100 MHz; MeOD) δ: 160.3, 158.8, 155.6, 150.6, 131.3, 132.4, 129.1, 123.4, 118.9, 114.8, 109.1, 107.2, 102.3, 71.1. *m*/*z* ESI-MS: [M + H]^+^ 260.09.

#### 4.1.19. (E)-2-Hydroxy-benzaldehyde-O-(3-hydroxybenzyl)-oxime **2a**

The crude was purified by trituration with hexane affording compound **2a** a white solid, 41% yield, m.p.: 105–108 °C; ^1^H NMR (400 MHz, DMSO-*d_6_*) δ: 9.91 (bs, 1H, -OH); 9.41 (bs, 1H, -OH); 8.41 (s, 1H, N=CH); 7.49 (dd, 1H, *J*_1_ = 2.0 Hz, *J*_2_ = 8.0 Hz, Ar); 7.25–7.21 (m, 1H, Ar); 7.16–7.12 (m, 1H, Ar); 6.88–6.78 (m, 4H, Ar); 6.69–6.67 (m, 1H, Ar); 5.05 (s, 2H, -CH_2_O). ^13^C-NMR (100 MHz; MeOD) δ: 157.3, 156.9, 150.6, 138.6, 130.8, 129.8, 129.1, 119.3, 119.1, 116.7, 115.9, 114.7, 114.62, 76.1. *m*/*z* ESI-MS: [M + H]^+^ 244.09.

#### 4.1.20. (E)-3-Hydroxy-benzaldehyde-O-(3-hydroxybenzyl)-oxime 2b

The crude was purified by trituration with hexane affording compound **2b** a white solid, 78% yield, m.p.: 84–86 °C; ^1^H NMR (400 MHz, DMSO-*d_6_*) δ: 9.59 (s, 1H, -OH); 9.41 (s, 1H, -OH); 8.20 (s, 1H, N=CH); 7.20 (t, 1H, *J* = 8.0 Hz, Ar); 7.17–7.13 (m, 1H, Ar); 7.03–6.99 (m, 2H, Ar); 6.81–6.78 (m, 3H, Ar); 6.70–6.67 (m, 1H, Ar); 5.05 (s, 2H, -CH_2_O). ^13^C-NMR (100 MHz; MeOD) δ: 159.5, 159.2, 150.9, 141.4, 135.6, 131.4, 131.0, 121.0, 120.6, 118.8, 116.6, 116.4, 114.7, 77.8. *m*/*z* ESI-MS: [M + H]^+^ 244.09.

#### 4.1.21. (E)-4-Hydroxy-benzaldehyde-O-(3-hydroxybenzyl)-oxime **2c**

The crude was purified by trituration with hexane affording compound **2c** as a white solid, 75% yield, m.p.: 125–127 °C; ^1^H NMR (400 MHz, MeOD) δ: 8.06 (s, 1H, N=CH); 7.44–7.42 (m, 2H, Ar); 7.17–7.13 (m, 1H, Ar); 6.85–6.83 (m, 2H, Ar); 6.81–6.76 (m, 2H, Ar); 6.72–6.69 (m, 1H, Ar); 4.88 (s, 2H, -CH_2_O). ^13^C-NMR (100 MHz; MeOD) δ: 160.5, 158.5, 150.3, 140.9, 130.3, 129.7, 124.9, 120.2, 116.5, 115.9, 115.6, 76.8. *m*/*z* ESI-MS: [M + H]^+^ 244.09.

#### 4.1.22. (E)-3,5-Dihydroxy-benzaldehyde-O-(3-hydroxybenzyl)-oxime **2d**

The crude was purified by trituration with hexane affording compound **2d** as a white solid, 64% yield, m.p.: 155–157 °C; ^1^H NMR (400 MHz, DMSO-*d_6_*) δ: 9.41 (s, 3H, -OH); 8.08 (s, 1H, N=CH); 7.16–7.12 (m, 1H, Ar); 6.78–6.76 (m, 2H, Ar); 6.70–6.67 (m, 1H, Ar); 6.45 (d, 2H, *J* = 2.4 Hz, Ar); 6.23 (t, 1H, *J* = 2.4 Hz, Ar); 5.03 (s, 2H, -CH_2_O). ^13^C-NMR (100 MHz; MeOD) δ: 159.8, 158.5, 150.4, 140.7, 135.6, 130.3, 120.3, 115.9, 115.6, 106.5, 105.2, 77.0. *m*/*z* ESI-MS: [M + H]^+^ 260.09.

#### 4.1.23. (E)-2,4-Didroxy-benzaldehyde-O-(3-hydroxybenzyl)-oxime **2e**

The crude was purified by trituration with hexane affording compound **2e** as a white solid, 77% yield, m.p.: 110–112 °C; ^1^H NMR (400 MHz, DMSO-*d_6_*) δ: 9.83 (s, 1H, -OH); 9.81 (s, 1H, -OH); 9.40 (s, 1H, -OH); 8.30 (s, 1H, N=CH); 7.29–7.27 (m, 1H, Ar); 7.16–7.11 (m, 1H, Ar); 6.78–6.76 (m, 2H, Ar); 6.69–6.66 (m, 1H, Ar); 6.28–6.26 (m, 2H, Ar); 4.99 (s, 2H, -CH_2_O). ^13^C-NMR (100 MHz; MeOD) δ: 161.8, 160.1, 1584, 152.2, 140.1, 132.6, 130.3, 120.3, 115.9, 115.8, 110.2, 108.6, 103.5, 77.10. *m*/*z* ESI-MS: [M + H]^+^ 260.09.

#### 4.1.24. (E)-3-Hydroxy-benzaldehyde-O-(4-hydroxybenzyl)-oxime **3b**

The crude was purified by trituration with hexane affording compound **3b** a white solid, 72% yield, m.p.: 88–91 °C; ^1^H NMR (400 MHz, MeOD) δ: 8.19 (s, 1H, N=CH); 7.25–7.21 (m, 2H, Ar); 7.19–7.16 (m, 1H, Ar); 7.06–7.04 (m, 1H, Ar); 7.02–6.99 (m, 1H, Ar); 6.81–6.88 (m, 1H, Ar); 6.77–6.74 (m, 2H, Ar); 5.03 (s, 2H, -CH_2_O). ^13^C-NMR (100 MHz; MeOD) δ: 158.8, 158.4, 149.9, 135.1, 131.2, 130.7, 129.7, 119.8, 118.0, 116.0, 113.9, 77.2. *m*/*z* ESI-MS: [M + H]^+^ 244.09.

#### 4.1.25. (E)-3,5-Dihydroxy-benzaldehyde-O-(4-hydroxybenzyl)-oxime **3d**

The crude was purified by trituration with hexane affording compound **3d** as a white solid, 75% yield, m.p.: 93–95 °C; ^1^H NMR (400 MHz, DMSO-*d_6_*) δ: 9.43 (s, 1H, -OH); 9.38 (s, 2H, -OH); 8.00 (s, 1H, N=CH); 7.19–7.17 (m, 2H, Ar); 6.74–6.71 (m, 2H, Ar); 6.44 (d, 2H, *J* = 2.0 Hz, Ar); 6.21 (t, 1H, *J* = 2.0 Hz, Ar); 4.96 (s, 2H, -CH_2_O). ^13^C-NMR (100 MHz; DMSO-*d_6_*) δ: 158.6, 157.2, 149.0 133.5, 130.1, 127.6, 114.9, 104.9, 104.2, 75.3. *m*/*z* ESI-MS: [M + H]^+^ 260.09.

#### 4.1.26. (E)-2,4-Dihydroxy-benzaldehyde-O-(4-hydroxybenzyl)-oxime **3e**

The crude was purified by trituration with hexane affording compound **3e** as a white solid, 67% yield, m.p.: 121–123 °C; ^1^H NMR (400 MHz, DMSO-*d_6_*) δ: 9.84 (s, 1H, -OH); 9.80 (s, 1H, -OH); 9.43 (s, 1H, -OH); 8.25 (s, 1H, N=CH); 7.28–7.26 (m, 1H, Ar); 7.20–7.18 (m, 2H, Ar); 6.75–6.72 (m, 2H, Ar); 6.29–6.27 (m, 2H, Ar); 4.95 (s, 2H, -CH_2_O). ^13^C-NMR (100 MHz; MeOD) δ: 161.7, 160.2, 158.6, 152.2, 132.7, 131.4, 129.4, 116.2, 110.4, 108.7, 103.6, 77.3. *m*/*z* ESI-MS: [M + H]^+^ 260.09.

### 4.2. Computational Details

In silico experiments were conducted on a multicore system (56 Intel Xeon E5-2660-v4@2.00GHz) equipped with two graphic cards (GPU) NVIDIA GeForce RTX 2070, using Ubuntu as the operating system, running Maestro Molecular Modelling suite release 2018 (Schrödinger, LLC, New York, NY, USA, 2018).

#### 4.2.1. Database and Protein Preparation

The chemical library was built using the drawing tools in the Maestro suite (Maestro release 2018, Schrödinger, LLC, New York, NY, USA, 2018). All the generated structures were minimized using MacroModel software (MacroModel release 2018, Schrödinger, LLC, New York, NY, USA, 2018) employing the force field OPLS3 [67]. To simulate the solvent effect, the GB/SA model was used, with “no cutoff” for non-bonded interactions. The PRCG method (5000 maximum iterations and threshold for gradient convergence = 0.001) was utilized. The resulting structures were treated by LigPrep software (LigPrep release 2018, Schrödinger, LLC, New York, NY, USA, 2018) to identify the most plausible ionization state at a physiological pH value (7.4 ± 0.2).

The three-dimensional structure of the enzyme (human SIRT1) was downloaded from the Protein Data Bank (PDB ID 5BTR [35]; crystal structure of SIRT1 in complex with resveratrol and an AMC-containing peptide, DOI: 10.2210/pdb5BTR/pdb) and imported into Maestro suite 2018. The protein structure was prepared by employing the protein preparation wizard protocol to obtain a reasonable starting structure for further in silico studies [43].

#### 4.2.2. Docking Studies

The library, prepared as described, was screened against the SIRT1 enzyme by Glide software (Glide release 2018, Schrödinger, LLC, New York, NY, USA, 2018) with the XP-scoring function and the SP-scoring function to perform a rescoring step, as previously described [12,43]. Because of the mechanism of activation of SIRT1 described for resveratrol and highlighted by the crystal structure in which resveratrol is bound to SIRT1 in three binding sites, we prepared the energy grids for docking, using the default value of the protein atom-scaling factor (1.0 Å), with a cubic box centered on crystallized resveratrol molecules. Following this procedure, we prepared a specific grid for each binding site found for resveratrol. After the grid generation, the library of compounds was docked into SIRT1, taking into consideration the three different binding sites of resveratrol. The docked poses considered for the post-docking minimization step totaled 1000, evaluating the Glide XP docking score (GlideScore). Remarkably, the XP technique was capable of correctly accommodating the resveratrol into the three distinct binding sites.

To improve the quality of the screening, we also assessed the ligand binding energies from the complexes derived by the molecular docking calculation. To this end, the Prime/MM-GBSA method available in Prime software (Prime release 2018, Schrödinger, LLC, New York, NY, USA, 2018) was used. This technique computes the variation between the free and the complex state of both the ligand and enzyme after energy minimization [43,68,69].

#### 4.2.3. Evaluation of Drug-like Profile

The in silico drug-like profile was determined using QikProp software (QikProp release 2018, Schrödinger, LLC, New York, NY, USA, 2018). Pan Assay Interference Compounds (PAINS) evaluation was performed by the FAFDrugs4 web server [70] (https://fafdrugs4.rpbs.univ-paris-diderot.fr/ (accessed on 15 February 2021)) as previously described [71].

### 4.3. Cell-Free Assay on Isolated SIRT1 Enzyme

SIRT1 activity was measured using a SIRT1 direct fluorescent screening assay kit (Cayman Chemical, Ann Arbor, MI, USA), according to the manufacturer’s protocol. Briefly, potential SIRT1 activators were freshly dissolved in DMSO (10^−^^2^ M) and diluted in the assay buffer up to a final concentration of 100 µM. Then, the reconstituted SIRT1 human recombinant enzyme, assay buffer, and selected compounds or vehicle (DMSO 1%) were added in triplicate to a 96-well plate. The SIRT1 activator resveratrol 100 µM [43] and the sirtuin inhibitor sirtinol 10 µM were used as positive and negative controls, respectively. The multi-well plate was incubated in the dark for 45 min at room temperature on a shaker. Then, 50 µL of a stop/developing solution was added to each well and incubated for 30 min at room temperature to allow the generation of a fluorescent reaction product. The fluorescence intensity was measured with the microplate reader EnSpire (PerkinElmer, Waltham, MA, USA) at λex = 350 nm and λem = 450 nm. The possible interference of compounds with the developer and/or fluorophore was evaluated before performing the assay. In a separate set of experiments, resveratrol and the most effective SIRT1 activator compound **3d** were tested also at lower concentrations (10 and 30 µM). Once the fluorescence values of the background (DMSO 1%) were subtracted, results were normalized considering the vehicle as 0% of SIRT1 activity and resveratrol 100 µM as 100%. Data are shown as the mean ± standard error of the mean (SEM). Statistical analysis included a one-way ANOVA followed by Bonferroni’s post hoc test, and statistical significance was set at *p* < 0.05. Analysis was performed with the software GraphPad Prism (version 5.0).

### 4.4. Cell Cultures

Human umbilical vein endothelial cells (HUVECs; Life Technologies, Carlsbad, CA, USA) were cultured at 37 °C in T-75 flasks in a CO_2_ (5%) incubator. At approximately 90% confluence, cells between passages 3 and 15 were used for the experimental procedures. The culture medium was composed of the basal medium (Medium 131; Life Technologies, USA) supplemented with antibiotics (100 U/mL penicillin and 100 mg/mL streptomycin), fetal bovine serum (FBS 10%; Merck KGaA, Germany) heated at 56 °C for 30 min, heparin from porcine intestinal mucosa (10 U/mL; Merck KGaA, Germany), L-glutamine (1%; Merck KGaA, Germany), basic fibroblast growth factor (bFGF; 5 ng/mL, PeproTech, Cranbury, NJ, USA), and epidermal growth factor (EGF; 10 ng/mL, PeproTech, USA).

### 4.5. Cell Viability Assay

Ninety-percent-confluent HUVECs were seeded at a density of 20,000 cells/well in a 96-well transparent plate. After 24 h, the culture medium was removed and cells were treated for 1 h with a DMSO solution of resveratrol (10, 30, and 100 µM), compound **3d** (10, 30, and 100 µM), or vehicle (DMSO 0.01%). Such concentrations have been selected based on previous experiments evaluating the potential effects of resveratrol against oxidative stress and angiogenesis in HUVECs [55,72,73].

Then, an aqueous solution of hydrogen peroxide (H_2_O_2_, 100 µM; ProFar, Milan, Italy) was incubated for 2 h to induce oxidative cell damage. At the end of treatment, a solution of the water-soluble tetrazolium salt WST-1 (Roche, Basel, Switzerland) was added (1:10) in the dark and the multi-well plate was placed at 37 °C in a CO_2_ incubator for 1 h. Cell viability was measured using the microplate reader EnSpire (PerkinElmer, Waltham, MA, USA) at λ = 495 nm.

In a separate set of experiments, a DMSO solution of the sirtuin inhibitor sirtinol (Sirt, 10 µM; Tocris, Bristol, UK) has been incubated for 1 h before adding the most effective concentration of resveratrol and compound **3d** to investigate the potential role of sirtuin in the preventive effects of both compounds.

Each experiment was performed in triplicate a minimum of four times (*n* = 12). Data were expressed as the percentage of absorbance measured in vehicle-treated cells (100%). Results are shown as mean ± standard error of the mean (SEM). Statistical analysis included a one-way ANOVA followed by Bonferroni’s post hoc test, and statistical significance was set at *p* < 0.05. Analysis was performed with the software GraphPad Prism (version 5.0).

### 4.6. Measurement of Intracellular ROS Production

HUVECs were seeded in a 96-well black plate at a density of 30,000 cells/well. Before seeding, the wells were pre-coated with an aqueous solution of gelatin (1%). After 24 h, the culture medium was replaced with a fresh medium containing a DMSO solution of resveratrol (10, 30, and 100 µM), compound **3d** (10, 30, and 100 µM), or the vehicle (DMSO 0.01%). Then, an aqueous solution of H_2_O_2_ (100 µM) was incubated for 2 h. At the end of treatment, the medium was removed and a freshly prepared solution of dihydroethidium (DHE 10 µM; Merck KGaA, Germany) dissolved in DMSO was added to each well. The plate was incubated in the dark at 37 °C for 30 min in a CO_2_ incubator to allow the probe to cross cell membranes and react with intracellular superoxide. Fluorescence values, corresponding to intracellular ROS production, were measured using the microplate reader EnSpire (PerkinElmer, USA) at λex = 500 nm and λem = 580 nm.

In a separate set of experiments, a DMSO solution of the sirtuin inhibitor sirtinol (Sirt, 10 µM) was incubated for 1 h before the tested compounds were added to investigate the potential role of sirtuins in the antioxidant effects of resveratrol and compound **3d**.

Each experiment was performed in triplicate a minimum of four times (*n* = 12). Data were expressed as the percentage of fluorescence measured in vehicle-treated cells (100%). Results are shown as mean ± standard error of the mean (SEM). Statistical analysis included a one-way ANOVA followed by Bonferroni’s post hoc test, and statistical significance was set at *p* < 0.05. Analysis was performed with the software GraphPad Prism (version 5.0).

## 5. Conclusions

In conclusion, in this work, we identified a new chemical class of SIRT1 activators, starting from the chemical structure of resveratrol. Among them, compound **3d** can be considered the synthetic derivative of greater pharmacological interest as it is endowed with antioxidant effects in the human endothelium, which result, at least in part, from its SIRT1-activating properties. Of note, even if compound **3d** appeared to be a weaker activator of the isolated SIRT1 enzyme than resveratrol, the preventive effects exhibited by both compounds in endothelial cells in terms of cell viability and ROS production were almost superimposable, suggesting a higher ability of compound **3d** to cross cell membranes and activate the intracellular target SIRT1. This is in line with the predicted physicochemical profile of **3d** for which we observed a better solvent-accessible surface along with a slightly better predicted intestinal adsorption (MDCK model) that is reflected in improved predicted human oral adsorption with respect to that found for resveratrol. However, in vivo models are needed to confirm our preliminary results on this promising resveratrol derivative and provide more precise conclusions about the potentially favorable pharmacokinetic profile of compound **3d**.

## Figures and Tables

**Table 1:** Substitution pattern of compounds of the in-house library **1a–e**, **2a–e**, **3b, d–e**, **4a–e, 5a–e, 6a–e,** and **7–9**.

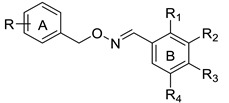					
Compound	R	R_1_	R_2_	R_3_	R_4_
**1a**	2-OH	OH	H	H	H
**1b**	2-OH	H	OH	H	H
**1c**	2-OH	H	H	OH	H
**1d**	2-OH	H	OH	H	OH
**1e**	2-OH	OH	H	OH	H
**2a**	3-OH	OH	H	H	H
**2b**	3-OH	H	OH	H	H
**2c**	3-OH	H	H	OH	H
**2d**	3-OH	H	OH	H	OH
**2e**	3-OH	OH	H	OH	H
**3b**	4-OH	H	OH	H	H
**3d**	4-OH	H	OH	H	OH
**3e**	4-OH	OH	H	OH	H
**4a**	2-OCH_3_	H	OH	H	OH
**4b**	2-OCH_3_	OH	OH	OH	H
**4c**	2-OCH_3_	H	OH	OH	H
**4d**	2-OCH_3_	OH	H	H	OH
**4e**	2-OCH_3_	OH	H	OH	H
**5a**	3-OCH_3_	H	OH	H	OH
**5b**	3-OCH_3_	OH	OH	OH	H
**5c**	3-OCH_3_	H	OH	OH	H
**5d**	3-OCH_3_	OH	H	H	OH
**5e**	3-OCH_3_	OH	H	OH	H
**6a**	4-OCH_3_	H	OH	H	OH
**6b**	4-OCH_3_	OH	OH	OH	H
**6c**	4-OCH_3_	H	OH	OH	H
**6d**	4-OCH_3_	OH	H	H	OH
**6e**	4-OCH_3_	OH	H	OH	H
**7**	2-Cl	H	OH	H	OH
**8**	3-Cl	H	OH	H	OH
**9**	4-Cl	H	OH	H	OH

**Table 2:** Top-ranked hits and their parameters obtained from in silico studies.

Cpd	GlideScore (kcal/mol)	ΔG_bind_ (kcal/mol)	SASA ^a^	QPlogP ^b^	QPlogS ^c^	QPPCaco ^d^	QPPMDCK ^e^	QPlogHERG ^f^	%HOA ^g^
**1d**	#site1 −7.34 #site2 −7.38 #site3 −7.26	#site1 −89.2 #site2 −69.1 #site3 −82.6	544	1.49	−2.88	276	131	−5.86	80
**2d**	#site1 −8.21 #site2 −7.59 #site3 −7.42	#site1 −89.7 #site2 −75.2 #site3 −83.1	565	1.42	−2.97	194	129	−5.94	78
**3b**	#site1 −7.86 #site2 −7.55 #site3 −7.29	#site1 −90.2 #site2 −76.6 #site3 −82.8	532	2.27	−3.21	622	397	−6.07	89
**3d**	#site1 −8.16 #site2 −7.68 #site3 −7.27	#site1 −89.4 #site2 −74.5 #site3 −81.9	545	1.40	−2.95	198	126	−5.95	81
**8**	#site1 −8.26 #site2 −7.22 #site3 −7.53	#site1 −90.6 #site2 −79.3 #site3 −83.7	556	2.73	−3.92	621	729	−5.99	94
**9**	#site1 −8.59 #site2 −7.74 #site3 −7.29	#site1 −91.4 #site2 −77.8 #site3 −82.1	557	2.69	−3.87	616	724	−5.98	95
**Rsv ^h^**	#site1 −7.12 #site2 −7.16 #site3 −7.21	#site1 −89.2 #site2 −64.7 #site3 −81.4	482	2.00	−2.81	279	123	−5.33	71

^a^ SASA predicted the total solvent accessible surface (range or recommended value for 95% of known drugs 300–1000); ^b^ QPlogP predicted octanol/water partition coefficient (range or recommended value for 95% of known drugs −2–6.5); ^c^ QPlogS predicted aqueous solubility in mol/dm^3^ (range or recommended value for 95% of known drugs −6.5–0.5); ^d^ QPPCaco predicted apparent Caco-2 cell permeability in nm/sec (range or recommended value for 95% of known drugs >500 great); ^e^ QPPMDCK predicted apparent MDCK cell permeability in nm/sec (range or recommended value for 95% of known drugs >500 great); ^f^ QPlogHERG predicted IC_50_ values for blockage of HERG K^+^ channels (range or recommended value for 95% of known drugs below −5); ^g^ %HOA predicted human oral absorption on 0 to 100% scale (range or recommended value for 95% of known drugs >80% high). Range or recommended values are reported in QikProp user manual; ^h^ resveratrol.

**Table 3:** Activity (%) of isolated SIRT1 enzyme in the presence of the reference activator resveratrol (100 µM), the sirtuin inhibitor sirtinol (10 µM), or the screened compounds (all 100 µM). Results are shown as mean ± SEM.

Compound	Concentration	SIRT1 Activity %
Resveratrol	100 µM	100.0 ± 2.7
Sirtinol	10 µM	−6.2 ± 4.1
**1d**	100 µM	1.1 ± 6.6
**2d**	100 µM	19.4 ± 3.4
**3b**	100 µM	23.0 ± 5.2
**3d**	100 µM	62.8 ± 5.6
**8**	100 µM	4.7 ± 8.5
**9**	100 µM	27.3 ± 5.2

## Data Availability

Not applicable.
